# Integrated Protein–Protein Interaction and Weighted Gene Co-expression Network Analysis Uncover Three Key Genes in Hepatoblastoma

**DOI:** 10.3389/fcell.2021.631982

**Published:** 2021-02-26

**Authors:** Linlin Tian, Tong Chen, Jiaju Lu, Jianguo Yan, Yuting Zhang, Peifang Qin, Sentai Ding, Yali Zhou

**Affiliations:** ^1^Department of Microbiology, Faculty of Basic Medical Sciences, Guilin Medical University, Guilin, China; ^2^Department of Urology, Shandong Provincial Hospital Affiliated to Shandong First Medical University, Jinan, China; ^3^Department of Urology, Shandong Provincial Hospital, Cheeloo College of Medicine, Shandong University, Jinan, China; ^4^Department of General Surgery, Shanghai Children’s Hospital, Shanghai Jiao Tong University, Shanghai, China; ^5^Key Laboratory of Tumor Immunology and Microenvironmental Regulation, Guilin Medical University, Guilin, China

**Keywords:** CCNA2, CDC20, CDK1, hepatoblastoma, PPI, WGCNA

## Abstract

Hepatoblastoma (HB) is the most common liver tumor in the pediatric population, with typically poor outcomes for advanced-stage or chemotherapy-refractory HB patients. The objective of this study was to identify genes involved in HB pathogenesis via microarray analysis and subsequent experimental validation. We identified 856 differentially expressed genes (DEGs) between HB and normal liver tissue based on two publicly available microarray datasets (GSE131329 and GSE75271) after data merging and batch effect correction. Protein–protein interaction (PPI) analysis and weighted gene co-expression network analysis (WGCNA) were conducted to explore HB-related critical modules and hub genes. Subsequently, Gene Ontology (GO) analysis was used to reveal critical biological functions in the initiation and progression of HB. Kyoto Encyclopedia of Genes and Genomes (KEGG) analysis showed that genes involved in cell cycle phase transition and the PI3K/AKT signaling were associated with HB. The intersection of hub genes identified by both PPI and WGCNA analyses revealed five potential candidate genes. Based on receiver operating characteristic (ROC) curve analysis and reports in the literature, we selected CCNA2, CDK1, and CDC20 as key genes of interest to validate experimentally. CCNA2, CDK1, or CDC20 small interfering RNA (siRNA) knockdown inhibited aggressive biological properties of both HepG2 and HuH-6 cell lines *in vitro*. In conclusion, we identified CCNA2, CDK1, and CDC20 as new potential therapeutic biomarkers for HB, providing novel insights into important and viable targets in future HB treatment.

## Introduction

Hepatoblastoma (HB) is caused by aberrant proliferation and/or differentiation of hepatic progenitor cell and represents a rare tumor that nevertheless accounts for most of liver tumors in infants and children (Allan et al., 2013). The majority of HB patients are diagnosed before 3 years of age, with a median age at diagnosis of 18 months (Spector and Birch, 2012). Over the past two decades, the incidence of HB has increased (Linabery and Ross, 2008; Bidwell et al., 2019), and HB now accounts for several cases per million per year in the pediatric population (Tulla et al., 2015). Combined modality therapy, including complete surgical resection and adjuvant cisplatin-based chemotherapy, has significantly improved the prognosis for HB. However, the prognosis of patients with advanced-stage or chemotherapy-refractory HB remains poor, with a 3-year event-free survival of less than 50% (Perilongo et al., 2004; Hiyama, 2014). Therefore, it is vital to identify biomarkers that may aid the discovery of new therapeutic strategies and thus improve the clinical management of advanced-stage or chemotherapy-refractory HB.

As an increasingly popular method to detect genome-wide gene expression, the combination of expression profile data and bioinformatics analysis has become an effective modality for the identification of potential biomarkers and key pathways in various diseases. In particular, in the context of tumor research, public databases have been widely used for the analysis of gene expression data. However, previous studies have so far focused mainly on the identification of differentially expressed genes (DEGs) between tumor and normal tissue, which cannot directly unveil the associations between genes (Langfelder and Horvath, 2008), rather than identifying complex relationships between genes. Protein–protein interaction (PPI) and/or weighted gene co-expression network analysis (WGCNA) are key methodologies that enable the identification of interactions between genes (Yuan et al., 2017) and can thus further our understanding of complex biological mechanisms (Murakami et al., 2017). It has been demonstrated that critical genes and pathways of several human tumors can be identified through PPI and/or WGCNA analyses (Shi et al., 2020; Wang et al., 2020).

In the context of HB, there have been two studies investigating gene regulatory networks and interconnectivity of functionally related genes so far (He et al., 2016; Aghajanzadeh et al., 2020). He et al. (2016) preliminarily identified genes, microRNAs, and the associated pathways involved in HB. More recently, Aghajanzadeh et al. (2020) screened the DEGs using GEO2R and conducted functional enrichment analyses by the EnrichR. They constructed PPI network of the up-regulated genes and then detected the significant modules. However, neither preprocessing of the raw data nor WGCNA was conducted in their study. In addition, their study included one dataset and lacked experimental verification of the results. Based on two publicly available datasets, the present study aimed to identify highly related differential genes and hub genes as potential biomarkers for HB. A variety of R packages were utilized for a better visualization of the results. We preprocessed raw data and conducted batch effect correction. We identified DEGs between HB and normal liver tissue and subsequently conducted PPI analysis in order to detect densely connected modules and candidate key genes from PPI network. Additionally, we conducted WGCNA to detect the module displaying the highest association with HB as well as key genes. Based on the intersection of hub modules obtained from PPI or WGCNA, biological functions and molecular signaling pathways involved in HB were explored via Gene Ontology (GO) and Kyoto Encyclopedia of Genes and Genomes (KEGG) analyses, respectively. These functional enrichment analyses were performed using the *clusterProfiler* R package. Moreover, we conducted an experimental verification of key genes by *in vitro* gene knockdown. Overall, our data may provide novel insights into important and viable targets for future HB treatment.

## Materials and Methods

### Data Retrieval and Extraction

HB-related data were obtained and downloaded from the Gene Expression Omnibus (GEO^1^) database portal using the keyword “hepatoblastoma.” The inclusion criteria for expression profile data were as follows: (a) the organism was *Homo sapiens*, (b) samples used for gene expression analysis included both HB tissue and normal liver tissue, (c) data for all samples were complete, and (d) HB and normal liver tissue samples could be clearly separated by principal component analysis (PCA). Only datasets that met all of the above criteria were included. Two datasets, GSE75271 (Sumazin et al., 2017) and GSE131329 (Kanawa et al., 2019), were therefore included for further analysis. GSE75271, consisting of 50 HB samples and five normal liver samples, was analyzed using GPL570 platform (Affymetrix Human Genome U133 Plus 2.0 Array), while GSE131329, consisting of 53 HB samples and 14 normal liver samples, was analyzed via GPL6244 platform (Affymetrix Human Gene 1.0 ST Array).

### Data Preprocessing and DEG Screening

Raw data files (^∗^.CEL) from GSE75271 and GSE131329 were downloaded and processed. Data from GPL570 and GPL6244 platforms were imported using R packages *affy* (Gautier et al., 2004) and *oligo* (Carvalho and Irizarry, 2010), respectively. The gene expression profile probe names were transformed to gene symbols and Entrez IDs using the *hgu133plus2.db* R Bioconductor package and the *hugene10sttranscriptcluster.db* R Bioconductor package, respectively. If one gene symbol corresponded to different probes, combined average levels were considered for gene expression values (Barrett et al., 2013). All raw data were processed using data filtering, a base 2 log transformation, and quantile normalization. The imput.knn function in the *impute* R Bioconductor package was used for data filtering. After data merging, the ComBat function in the *sva* R package (Leek et al., 2012) was used for batch effect correction, and the results were verified by PCA. DEGs between HB and normal liver tissue samples were detected using the *limma* R package (Ritchie et al., 2015), with the following cut-off criteria for significance: adjusted *P* < 0.05 and |log2FC| > 1.

### Functional Gene and Pathway Enrichment Analysis

In order to explore the functional annotation of candidate genes, GO terms and KEGG pathway analyses were performed using the *clusterProfiler* R package (Yu et al., 2012). GO terms included biological process (BP), cellular component (CC), and molecular function (MF). Adjusted *P* values below 0.05 were deemed significantly enriched.

### PPI Network Establishment

We constructed a PPI network using the Search Tool for the Retrieval of Interacting Genes (STRING) online database (Szklarczyk et al., 2015), with an interaction score >0.4 set as the cut-off value. Subsequently, the Cytoscape software was utilized to visualize the PPI network (Shannon et al., 2003). The Molecular Complex Detection (MCODE) (Bandettini et al., 2012) plugin in Cytoscape was applied in order to extract densely connected modules from PPI network, with degree cut-off = 2, node score cut-off = 0.2, *K*-score = 2, and max depth = 100. The Cytoscape plugin CytoHubba (Chin et al., 2014) was utilized for the identification of key genes from the PPI analysis. We extracted the top 20 genes from both approaches, and the intersecting genes of all four approaches of CytoHubba ranking were deemed as hub genes. The four approaches of CytoHubba ranking used here were maximal clique centrality (MCC), edge percolated component (EPC), maximum neighborhood component (MNC), and node connect degree.

### WGCNA

We constructed an unsigned weighted gene co-expression network using the *WGCNA* R package (Langfelder and Horvath, 2008). After data merging, batch effect correction, and exclusion of outlier samples, the complete gene expression matrix contained 8,204 genes across 116 samples. An expression matrix of 2,051 genes with the top 25% highest variance was used for WGCNA. We conducted hierarchical clustering of samples to remove outliers with a cut-off value of 80 to produce two stable clusters. Then, the soft threshold power β was determined in order to ensure a scale-free network. The resulting Pearson correlation matrix was converted to adjacency matrix via the power function, followed by transformation into a topological overlap matrix (TOM). The TOM was used to calculate corresponding dissimilarity. We carried out hierarchical clustering in order to cluster similar genes into the same module. The dynamic cutting algorithm was then used to detect the gene modules. Subsequently, we clustered the eigengenes according to the relationship and merged them into modules with the association >0.75. Module–trait association between each module and the phenotype was evaluated based on Pearson correlation. For each gene, module membership (MM) was characterized according to the association between module eigengene (ME) and its expression level. The association between gene expression and clinical phenotype represented gene significance (GS). After identifying a module of interest, GS and MM for each gene were computed in the given module. Finally, we performed GO and KEGG pathway analyses to illustrate potential biological functions of the identified module.

### Identification and Verification of Critical Genes

Key genes were closely correlated genes in one module with a MM > 0.8 and a GS > 0.2. For subsequent analysis, intersecting genes identified from both PPI and the most significant modules were assessed. Based on GSE75271 and GSE131329 datasets, the expression values of key genes between HB and normal liver tissue samples were then compared.

### Reagents and Antibodies

FBS (cat. no. 10099141), DMEM (cat. no. 11995065), PBS (cat. no. 10010023), and 0.25% Trypsin-EDTA (cat. no. 25200072) were purchased from Gibco (Grand Island, NY). Antibodies against CDK1 (cat. no. ab133327), CCNA2 (cat. no. ab181591), and β-actin (cat. no. ab8226) were obtained from Abcam (Cambridge, MA, United States). Antibodies against CDC20 (cat. no. 4823) and GAPDH (cat. no. 5174S) were procured from CST (Beverly, MA, United States).

### Cell Culture

Human HB cell lines (HepG2 and HuH-6) were purchased from Shanghai Institutes for Biological Sciences, Chinese Academy of Sciences. Cells were cultured in DMEM supplemented with 10% FBS at 37°C/5% CO_2_.

### Western Blot Assay

Total proteins were extracted from HepG2 or HuH-6 cells using the RIPA buffer supplemented with a protease inhibitor cocktail. Lysates were separated using sodium dodecyl sulfate polyacrylamide gel electrophoresis and transferred onto a polyvinylidene fluoride membrane. The membrane was then blocked for 1 h using western blocking buffer and subsequently incubated using a primary antibody, followed by incubation for 2 h with IgG HRP-conjugated secondary antibody (Jackson ImmunoResearch, PA, United States). Proteins were detected using ChemiDoc-It system (Tanon, Shanghai, China). Band intensities were assessed using ImageJ. GAPDH or β-actin served as the loading control.

### Small Interfering RNA

Small interfering RNAs (siRNAs) targeting CDK1, CCNA2, or CDC20, as well as non-targeting control siRNAs, were obtained from RiboBio (Guangzhou, China). siRNAs were transfected into HepG2 or HuH-6 cell lines according to the manufacturer’s guidelines using Lipofectamine 2000. Transfection efficiency was confirmed via western blot (WB) 2 days after siRNA transfection.

### Colony Formation Assay

HB cells were cultured in six-well plates containing media supplemented with 10% FBS to a density of 3 × 10^3^ cells/well. The culture media were replaced by media containing 5% FBS the following day, and cells were cultured for 2 weeks. This step was followed by paraformaldehyde (PFA) fixing and staining with crystal violet. Subsequently, photos were taken. Cells were subsequently fixed using PFA, stained with crystal violet, and microscopic images were acquired.

### Cell Viability Assay

Cell viability was assessed using Cell Counting Kit-8 (CCK-8, Dojindo, Japan). Briefly, cells were seeded into 96-well plates (1 × 10^3^ cells per well) and cultured for 4 h until adherence. The CCK-8 agent was added in each well at the indicated time-point, and the optical density at 450 nm was assessed after 1 h using a plate reader.

### Transwell Invasion Assay

The transwell invasion assay was carried out using six-well plates containing transwell inserts (8-μm pore size; BD Biosciences) according to the manufacturer’s guidelines. Matrigel purchased from BD Biosciences was added to serum-free media, transferred to the top chamber, and incubated for 5 h. Subsequently, cells were cultured in the top chamber supplemented with serum-free medium. The lower chamber was supplemented with 10% FBS, and cells were removed from the top chamber after 36-h incubation. For quantification of the cells in the lower chamber, membranes were PFA-fixed, stained with crystal violet, and invading cells were quantified using microscopy image analysis.

### Statistical Analysis

Data analysis was carried out using R (version 3.6.3) and GraphPad Prism (version 8.0.1). Gene expression levels between HB and normal liver tissue samples were compared using the Student’s *t*-test. To evaluate the predictive value of each hub gene for the distinction between HB and normal liver tissue, we applied the receiver operating characteristic (ROC) curve. An area under curve (AUC) > 0.90 and *P* < 0.05 indicated statistical significance.

## Results

### DEG Screening and Functional Annotation

**FIGURE 1:**
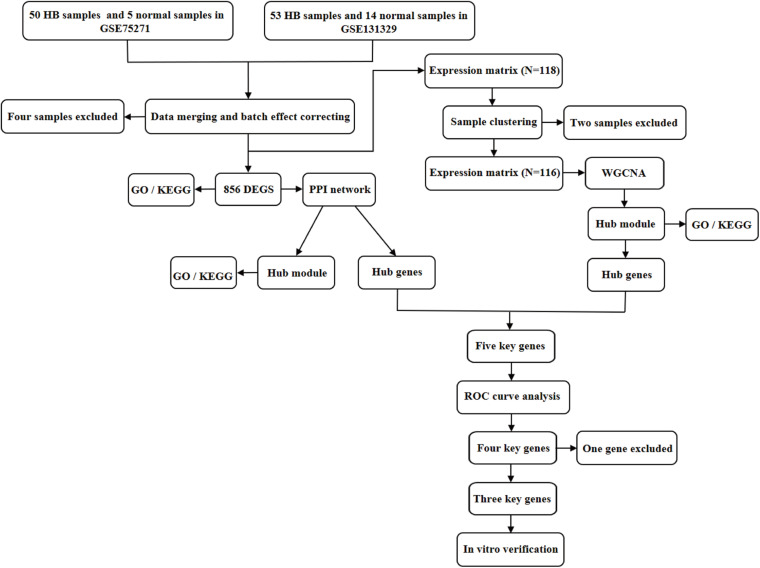
Flowchart illustrating the study design. DEGs, differentially expressed genes; GO, Gene Ontology; KEGG, Kyoto Encyclopedia of Genes and Genomes; PPI, protein–protein interaction; WGCNA, weighted gene co-expression network analysis; ROC, receiver operating characteristic.

**FIGURE 2:**
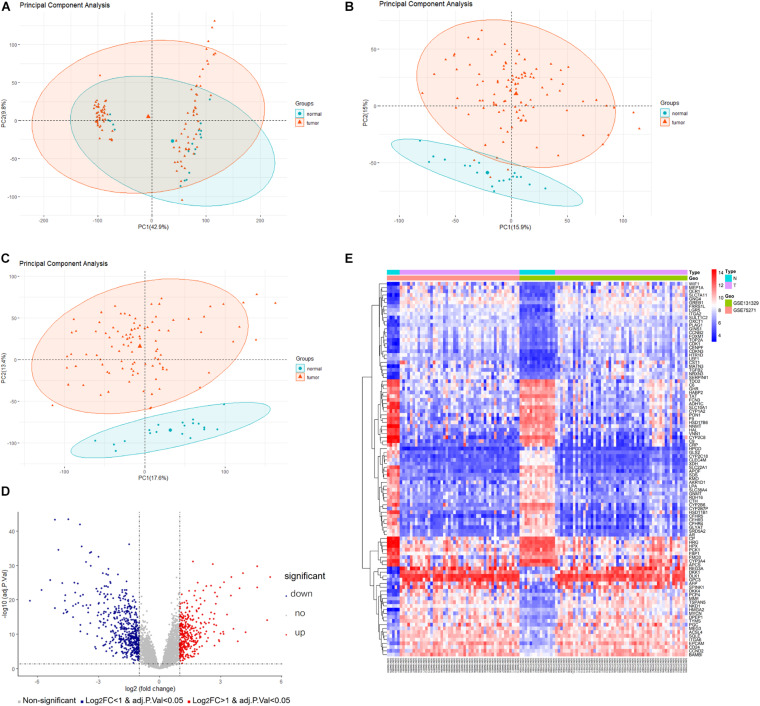
Data preprocessing and DEG analysis of the GSE75271 and GSE131329 datasets. Principal component analysis indicating the overall profiles of two datasets **(A)** before and **(B)** after batch effect correction and data merging. **(C)** Principal component analysis after removal of outlier samples. **(D)** Volcano plots visualizing DEGs between HB and normal liver tissue samples from the two datasets. Red points represent up-regulation, while blue points indicate down-regulation; gray points represent normal expression. **(E)** Heatmap of the top 50 up-regulated and top 50 down-regulated DEGs with *P* value <0.05 and logFC > 1. Red points represent up-regulation; blue points indicate down-regulation. DEG, differentially expressed gene.

**FIGURE 3:**
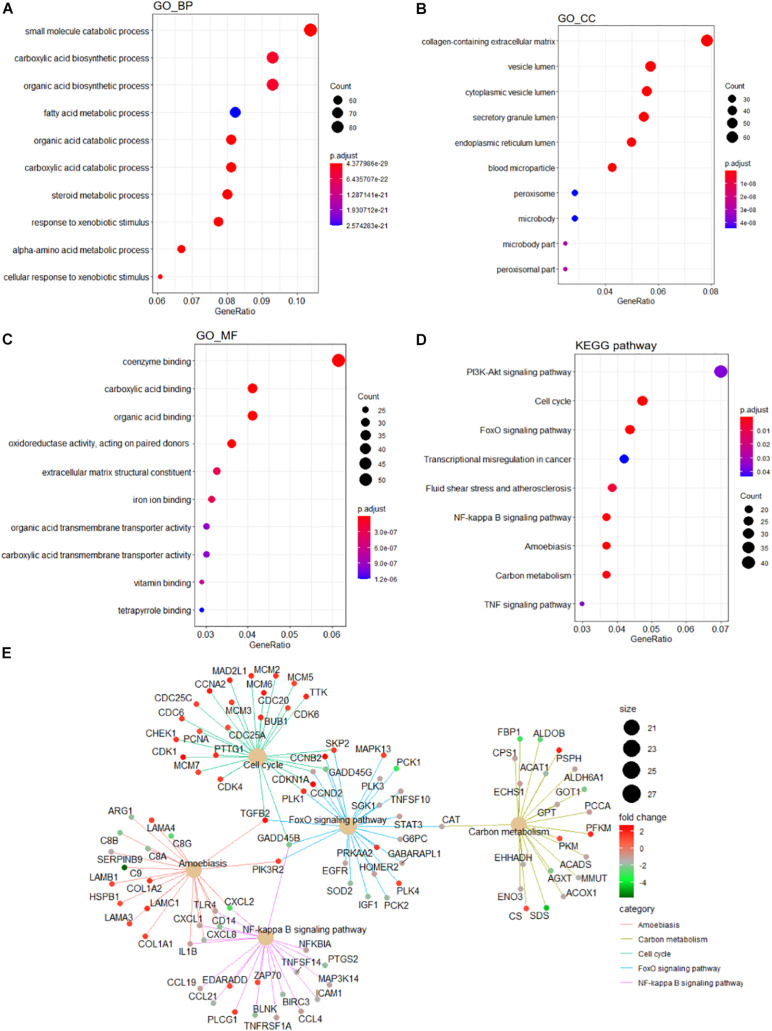
Functional enrichment analyses of the DEGs. GO analysis containing **(A)** BP terms, **(B)** CC terms, and **(C)** MF terms. **(D)** KEGG pathway analysis of the DEGs. **(E)** The cnetplot of KEGG pathways showing genes enriched in different pathways. The symbol adjacent to nodes represents the specific gene. The color bar represents the fold change of genes in the respective pathways. DEGs, differentially expressed gene; GO, Gene Ontology; KEGG, Kyoto Encyclopedia of Genes and Genomes; BP, biological process; CC, cellular component; MF, molecular function.

A detailed outline of our study is summarized in Figure 1. For our analysis, we combined two publicly available microarray gene expression datasets of HB and normal liver tissue samples. We carried out PCA to visualize data before and after batch effect correction, during which four outlier samples (GSM1948577, GSM1948562, GSM1948566, and GSM3770543) were removed (Figures 2A–C), resulting in a total of 99 HB samples and 19 normal liver samples after data preprocessing and quality control. We applied a filtering step (*P* value < 0.05 and |log2FC| > 1) for the identification of DEGs, which resulted in a total of 856 DEGs. Among these DEGs, 350 were up-regulated, while 506 were down-regulated, with a volcano plot presented in Figure 2D. The heatmap of the top 100 genes is shown in Figure 2E. We conducted GO and KEGG pathway analyses to elucidate the biological functions and potential signaling pathways these genes may be involved in. GO analysis suggested that DEGs predominantly consisted of genes involved in small molecule catabolic processes, the collagen-containing extracellular matrix, and coenzyme binding (Figures 3A–C). KEGG analysis identified enrichment for PI3K-AKT signaling, cell cycle, and FoxO signaling (Figure 3D). Critical pathways, including the cell cycle, FoxO pathway, NF-kappa B signaling pathway, amoebiasis, and carbon metabolism, are presented along with their related genes in Figure 3E. For the term cell cycle, all enriched DEGs were up-regulated with the exception of GADD45B and GADD45D. It should be noted that we observed two genes concurrently enriched in three critical pathways: TGFB2 was enriched in the cell cycle, FoxO signaling, and amoebiasis pathways, while GADD45B was enriched in the cell cycle, FoxO signaling, and NF-kappa B signaling pathways.

### PPI Network Establishment and Module Analyses

**FIGURE 4:**
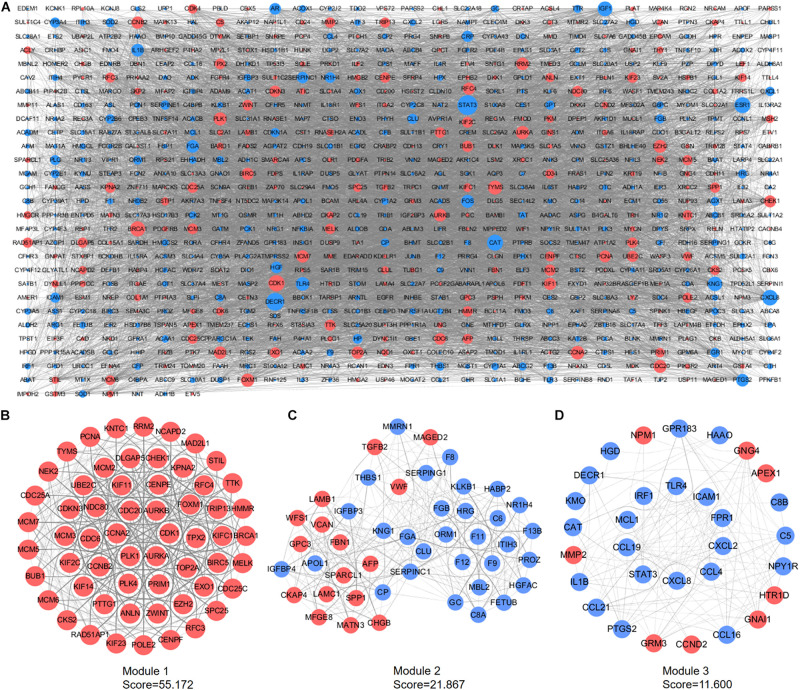
PPI network construction and module analyses. **(A)** PPI network of DEGs was constructed in Cytoscape. Red points represent up-regulated genes, while blue points represent down-regulated genes. The node size depends on the degree of node connectivity; edges indicate straight associations. **(B)** Module 1 contains 59 nodes and 1,600 edges. **(C)** Module 2 contains 46 nodes and 492 edges. **(D)** Module 3 includes 31 nodes and 174 edges. Red nodes represent up-regulated genes; blue nodes represent down-regulated genes. DEG, differentially expressed gene; PPI, protein–protein interaction.

**FIGURE 5:**
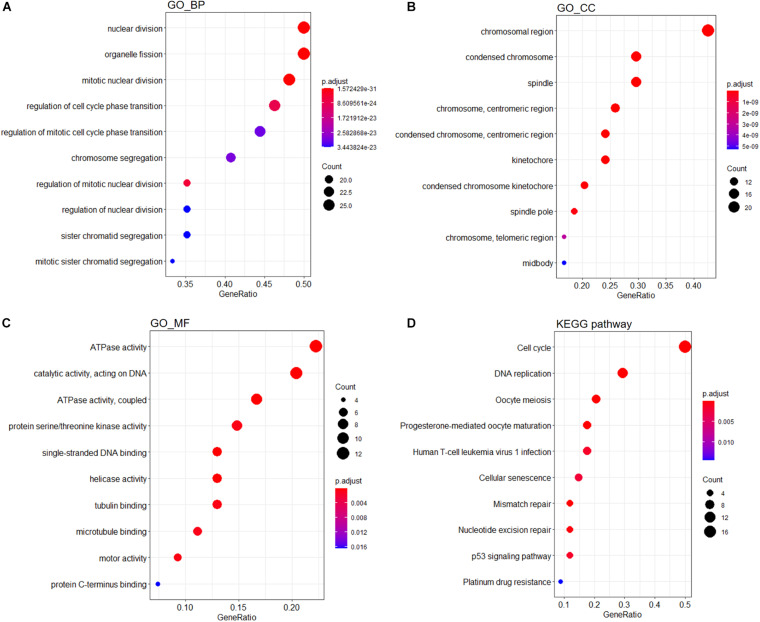
Functional enrichment analyses of genes from module 1. GO analysis containing **(A)** BP, **(B)** CC, and **(C)** MF terms. **(D)** KEGG analysis of significantly enriched pathways of genes in module 1. GO, Gene Ontology; KEGG, Kyoto Encyclopedia of Genes and Genomes; BP, biological process; CC, cellular component; MF, molecular function.

A PPI network containing 791 nodes and 9,054 edges was conducted using the Cytoscape software based on results of the STRING online database (Figure 4A). All four methods within the CytoHubba plugin were adopted, and the top 20 genes of each method were listed (Table 1). The intersecting genes that were concurrently listed in the four methods were regarded as hub genes (AURKA, AURKB, CDK1, CCNA2, CDC20, and PLK1) for PPI analysis. Nineteen clusters were obtained after module analysis using the MCODE plugin of Cytoscape, and we selected the top three modules as hub modules based on MCODE scores (Figures 4B–D). Notably, all six hub genes were found in module 1, which played an essential role in the constructed PPI network. Specifically, module 1 contained 59 nodes and 1,600 edges and had the highest MCODE score (55.172) of all modules. Another notable observation from module analysis was that all genes from module 1 exhibited up-regulation. Subsequently, we conducted GO and KEGG analyses of genes in module 1 using the R *clusterProfiler* package. For BP within the GO analysis, we found that genes in module 1 played a critical role in nuclear division, organelle fission, cell cycle transition, mitotic cell cycle transition, as well as chromosome segregation (Figure 5A). For CC within the GO analysis, we found that up-regulated genes were significantly enriched in the chromosomal region, condensed chromosome, and spindle (Figure 5B). The MF of GO analysis showed that genes were associated with ATPase activity, catalytic activity, action on DNA, protein serine/threonine kinase activity, and single-stranded DNA binding (Figure 5C). KEGG analysis showed that genes from module 1 were enriched for the cell cycle, DNA replication, as well as oocyte meiosis pathways (Figure 5D).

**TABLE 1:** Hub genes identified using the Cytohubba plugin (Cytoscape).

Category	Ranking methods in the CytoHubba plugin			
	MCC	EPC	MNC	Degree
1	*KIF11*	*KIF11*	***AURKA***	***AURKA***
2	*RRM2*	*RRM2*	***AURKB***	***AURKB***
3	***AURKA***	***AURKA***	*FOXM1*	*TLR4*
4	*TTK*	***AURKB***	***CDK1***	*FOXM1*
5	***AURKB***	*MAD2L1*	***CCNA2***	*DECR1*
6	*MAD2L1*	*FOXM1*	*EZH2*	***CDK1***
7	*DLGAP5*	*TOP2A*	*TYMS*	***CCNA2***
8	*TOP2A*	***CDK1***	***CDC20***	*EZH2*
9	***CDK1***	***CCNA2***	***PLK1***	*TYMS*
10	***CCNA2***	*TYMS*	*BRCA1*	*KNG1*
11	*CCNB2*	*CCNB2*	*TLR4*	*IGF1*
12	*UBE2C*	*UBE2C*	*DECR1*	***CDC20***
13	***CDC20***	***CDC20***	*KNG1*	*FOS*
14	*BIRC5*	***PLK1***	*IGF1*	***PLK1***
15	***PLK1***	*MCM7*	*FOS*	*EGFR*
16	*MELK*	*CDC6*	*EGFR*	*STAT3*
17	*KIF23*	*TPX2*	*STAT3*	*ESR1*
18	*CDC6*	*RFC4*	*ESR1*	*CXCL8*
19	*TPX2*	*BRCA1*	*CXCL8*	*CAT*
20	*BUB1*	*CHEK1*	*CAT*	*BRCA1*

*MCC, maximal clique centrality; EPC, edge percolated component; MNC, maximum neighborhood component; Degree, node connect degree. The bold values represent the genes that appear in all four ranking methods used here.*

### WGCNA and Hub Module Identification

**FIGURE 6:**
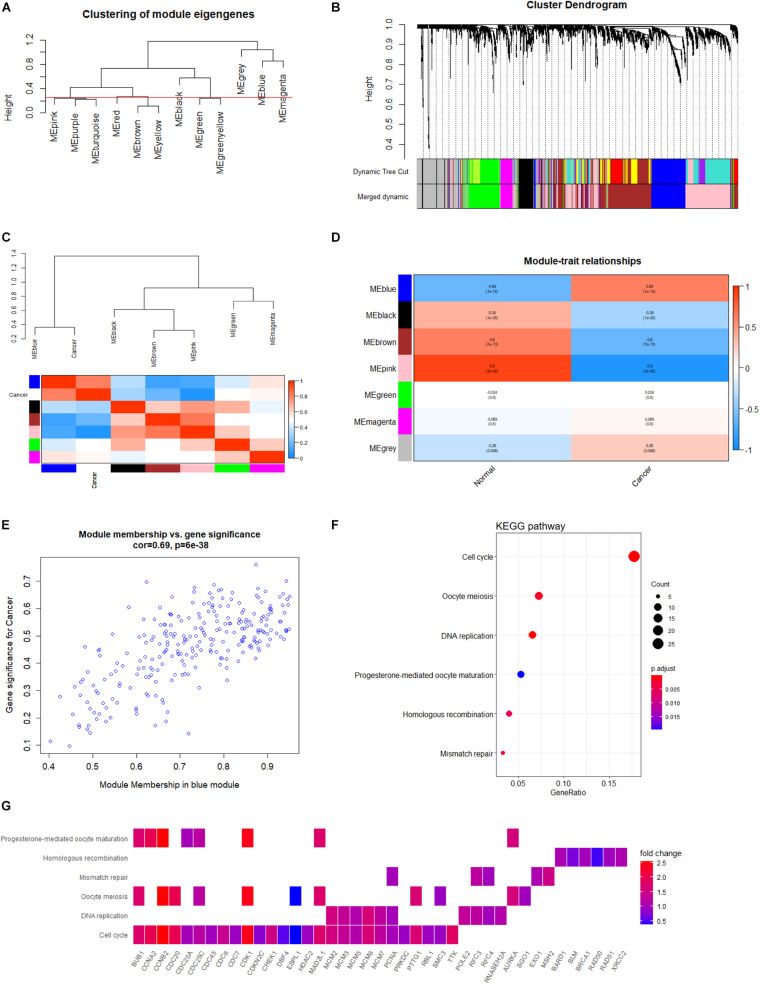
Co-expression network analysis based on WGCNA. **(A)** Clustering of module eigengenes with a threshold of 0.25 height to identify similar modules. **(B)** Identification of HB-specific modules. Each branch represents an expression module of a highly interconnected groups of genes; each color indicates a corresponding co-expression module. **(C)** Heatmap of the eigengene network indicates correlations between different modules; tightly connected modules are clustered together. **(D)** Heatmap of associations among module eigengenes in normal liver and HB samples. **(E)** Scatter plots highlighting the association between GS and MM based on genes from the blue module. **(F)** KEGG analysis of significantly enriched pathways based on genes from blue module. **(G)** Heatmap of specific genes associated with each enriched key pathway. WGCNA, weighted gene co-expression network analysis; HB, hepatoblastoma; GS, gene significance; MM, module membership; KEGG, Kyoto Encyclopedia of Genes and Genomes.

During sample clustering, two samples were regarded as outliers and thus excluded (GSM1948574 and GSM3770517; Supplementary Figure 1A). Besides, we identified β = 10 and *R*^2^ = 0.88 as the optimal soft threshold parameters to guarantee a scale-free network (Supplementary Figures 1B,C). We set clustering height cut-off to 0.25 in order to merge similar modules, which resulted in seven modules (Figure 6A). Specifically, blue, black, brown, pink, green, and magenta modules were identified as significant modules (Figure 6B). The blue module containing 259 genes appeared to be the most relevant module involved in HB. The top 100 genes of the blue module, ranked by gene significance for cancer, are listed in Supplementary Table 1. Subsequently, the module eigengenes and associations between eigengenes and sample types were computed. The module eigengene dendrogram was plotted, and the seven modules were divided into two clusters. Similar results were obtained from eigengene network heatmap (Figure 6C). Interestingly, the blue module was not only located close to cancer but also had a markedly positive association with cancer, meaning that genes in the blue module may be essential for tumor progression. Moreover, module–trait relationship analysis confirmed the highly positive correlation between the blue module and cancer (*r* = 0.64, *P* = 1e-14) (Figure 6D). When focusing on the blue module (Figure 6E), we substantiated a significantly positive association between MM and GS (*r* = 0.64, *P* = 6e-38). Consequently, the blue module was chosen for functional enrichment analysis, during which we aimed to elucidate potential biological processes involved in HB.

### GO and KEGG Functional Enrichment Analyses of the Blue Module

**FIGURE 7:**
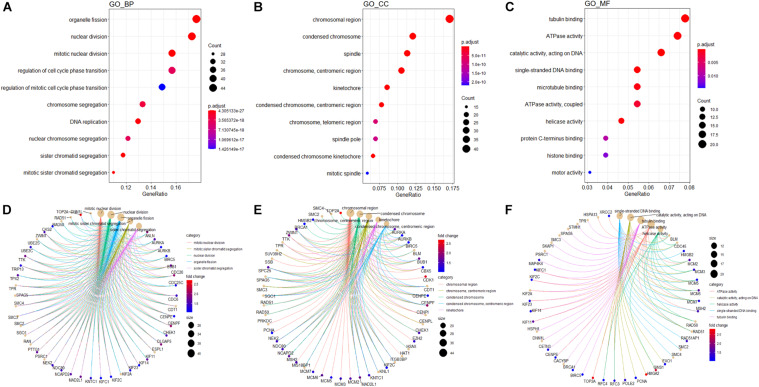
GO analysis of genes from blue module. The significant GO BP **(A)**, CC **(B)**, and MF **(C)** terms after enrichment analysis of genes from the WGCNA blue module. Cnetplot indicating specific genes associated with enriched GO BP **(D)**, CC **(E)**, or MF **(F)** terms; the symbol adjacent to the node represents the specific gene. GO, Gene Ontology; BP, biological process; CC, cellular component; MF, molecular function.

In order to explore potential genes and pathways associated with HB growth, we conducted GO and KEGG analyses on the blue module identified by WGCNA. KEGG analysis indicated that genes in the blue module were markedly enriched for the cell cycle, oocyte meiosis, and DNA replication pathways (Figure 6F). Key pathways and their associated genes are shown in the heatmap (Figure 6G). Additionally, GO analysis demonstrated that genes in the blue module were primarily associated with organelle fission, nuclear division, chromosomal region, tubulin binding, and ATPase activity (Figures 7A–C). For the description of functionally enriched GO clusters, we utilized cnetplots to highlight the relationships between genes and critical pathways (Figures 7D–F).

### Selection and Verification of Key Genes

**FIGURE 8:**
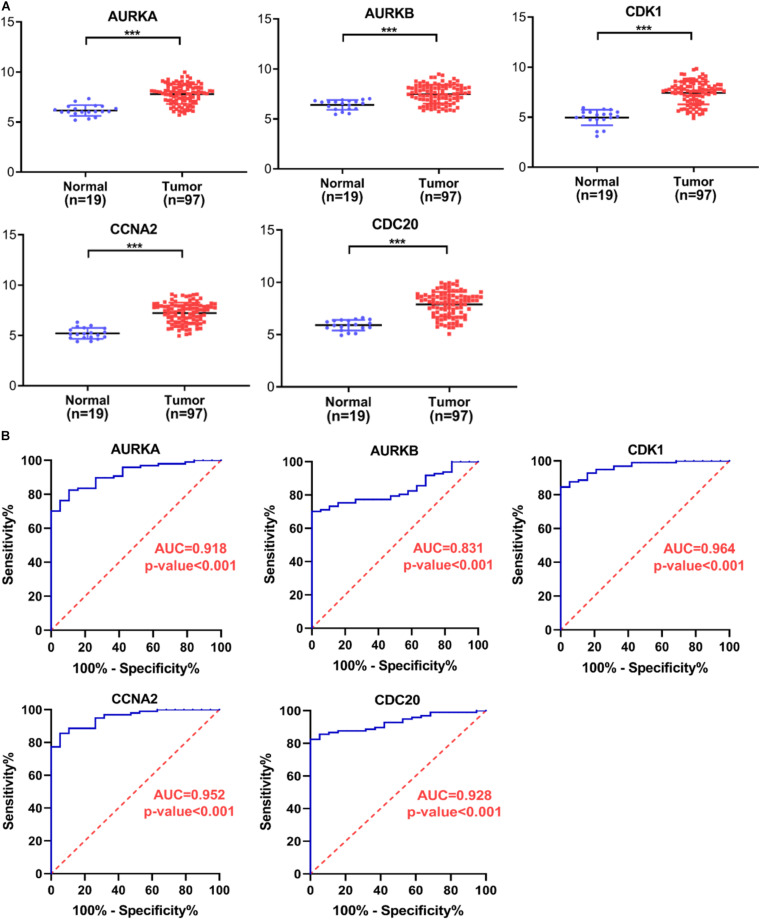
Verification of hub gene expression levels and ROC curve analysis. **(A)** The expression levels of AURKA, AURKB, CDK1, CCNA2, and CDC20 mRNAs were markedly up-regulated in HB samples relative to normal liver samples. **(B)** ROC curve analysis of AURKA, AURKB, CDK1, CCNA2, and CDC20. ROC, receiver operating characteristic; AUC, area under the curve. ***P < 0.001.

Several key genes identified using PPI analysis were also included in the WGCNA blue module, with the intersecting genes being AURKA, AURKB, CDK1, CCNA2, and CDC20. The scoring of each hub gene in PPI and WGCNA is summarized in Table 2. For further validation of these potential key genes, we compared their expression values between HB and normal liver samples in the GSE75271 and GSE131329 datasets. Expression levels of these five key genes were markedly elevated in HB samples compared with normal liver samples (Figure 8A). ROC curve was utilized to evaluate the predictive value of each hub gene for the distinction between HB and normal liver tissue. The AUC of expression levels for four of the genes exceeded 0.90 in the ROC analysis (Figure 8B). Specifically, the AUC was 0.918 (95% CI, 0.865–0.970) for AURKA, 0.964 (95% CI, 0.933–0.994) for CDK1, 0.952 (95% CI, 0.915–0.990) for CCNA2, and 0.928 (95% CI, 0.882–0.973) for CDC20. A literature search revealed AURKA as a previously reported oncogenic gene in HB, and elevated expression levels of AURKA have been associated with an advanced COG stage as well as metastasis (Zhang et al., 2018; Tan et al., 2020). However, the role of CDK1, CCNA2, or CDC20 in HB growth has not been reported to date, and thus, we selected these three genes for subsequent experimental validation.

**TABLE 2:** Scores of five intersecting hub genes using different ranking methods in PPI. and WGCNA.

Entrez ID	Gene Symbol	PPI				WGCNA			
		MCC	EPC	MNC	Degree	GS	*p*.GS	MM	*p*.MM
6790	*AURKA*	9.22E + 13	96.26	83	84	0.549	1.64E-10	0.850	1.37E-33
9212	*AURKB*	9.22E + 13	93.997	80	80	0.415	3.54E-06	0.806	1.00E-27
983	*CDK1*	9.22E + 13	98.24	101	102	0.644	6.22E-15	0.950	1.52E-59
890	*CCNA2*	9.22E + 13	97.444	85	86	0.612	2.75E-13	0.934	4.36E-53
991	*CDC20*	9.22E + 13	97.548	82	82	0.559	6.80E-11	0.851	1.06E-33

*PPI, protein–protein interaction; MCC, maximal clique centrality; EPC, edge percolated component; MNC, maximum neighborhood component; Degree, node connect degree; GS, gene significance with cancer; MM, module membership; *p*.GS, *p* value of gene significance with cancer; *p*.MM, *p* value of module membership.*

### CDK1, CCNA2, or CDC20 Knockdown Inhibits Proliferative, Migrative, and Invasive Capacities of HB Cell Lines

**FIGURE 9:**
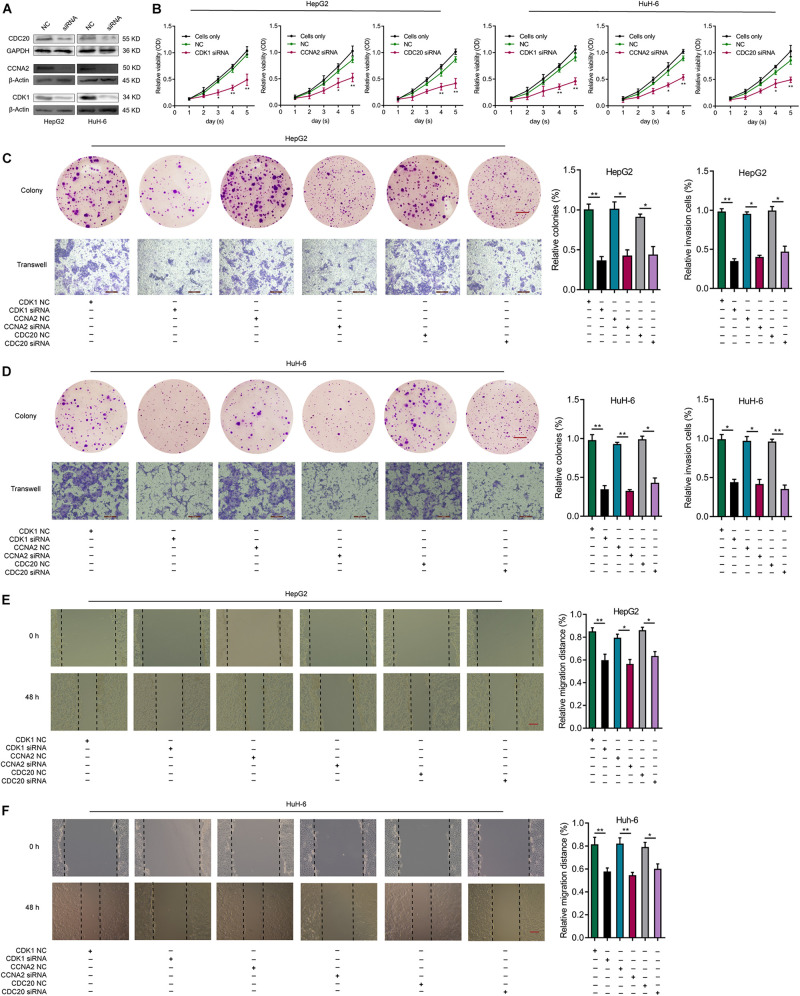
Knockdown of CDK1, CCNA2, or CDC20 inhibits proliferative, migrative, and invasive capacities of HB cells *in vitro*. **(A)** WB analysis confirm the knockdown efficiency of CDK1, CCNA2, or CDC20 2 days after transfection with siRNAs for CDK1, CCNA2, or CDC20. **(B)** The CCK-8 assay illustrates the proliferative capacity of HB cells after siRNA transfection. After siRNA transfection of HepG2 **(C)** or HuH-6 **(D)** cells, the proliferative and invasive capacities of the respective cell lines were evaluated by colony formation assays (scale bars, 8 mm) and transwell invasion assays (scale bars, 200 μm), respectively. **(E,F)** Wound healing assay (scale bars, 500 μm) results that indicate the migrative capacities of HepG2 **(E)** or HuH-6 **(F)** cells after transfection with siRNA. *P < 0.05, **P < 0.01. ROC, receiver operating characteristic; HB, hepatoblastoma; WB, western blot; siRNAs, small interfering RNAs; CCK-8, Cell Counting Kit-8.

In order to investigate the influence of CDK1, CCNA2, or CDC20 expression in HB cells, we knocked down CDK1, CCNA2, or CDC20 in HepG2 and HuH-6 cells via siRNAs. The knockdown efficiency of each hub gene was validated by WB analysis (Figure 9A). After transfection of CDK1-siRNA into HepG2 and HuH-6 cell lines, the effect of CDK1 knockdown on cell proliferation was explored using both a CCK-8 assay (Figure 9B) and a colony formation assay (Figures 9C,D). These assays indicated a significantly lower proliferative ability of the CDK1-siRNA group compared to the control siRNA group. Similar effects were observed for CCNA2 or CDC20 knockdown in HB cells (Figures 9B–D). Next, we evaluated the effect of CDK1, CCNA2, or CDC20 knockdown on the invasive ability of HB cells using a transwell invasion assay (Figures 9C,D), which revealed a significantly decreased rate of the relative invasive cells relative to controls for all three knockdown models. Lastly, wound-healing assays revealed that CDK1, CCNA2, or CDC20 siRNA knockdown groups exhibited a markedly lower relative migration distance than the control did (Figures 9E,F). Taken together, these results demonstrated that knockdown of CDK1, CCNA2, or CDC20 inhibited proliferative, migratory, and invasive capabilities in both HepG2 and HuH-6 cell lines.

## Discussion

HB is the most common liver tumor in the pediatric population, and its incidence has been consistently increasing in the last years. Surgical resection and adjuvant cisplatin-based chemotherapy may severely affect the health-related quality of life of HB patients and their families, and the therapeutic efficacy in patients with advanced-stage or chemotherapy-refractory HB is unsatisfactory. Therefore, further exploring the molecular mechanisms of HB is essential for early diagnosis and better treatment strategy.

The results of our study showed that DEGs between HB and normal liver tissue samples were primarily associated with PI3K-AKT signaling, cell cycle, and FoxO signaling. Forty DEGs were associated with PI3K-AKT signaling, indicating that these genes may be critical for HB growth. Indeed, a previous study reported that inhibition of the PI3K/AKT signaling pathway resulted in suppressed proliferation and increased apoptosis of HB cells (Hartmann et al., 2009). Moreover, FoxO signaling has been reported as a key signaling pathway closely associated with PI3K/AKT signaling in many human tumors (Farhan et al., 2017), and inhibition of FoxO signaling has been shown to lead to cell cycle arrest, apoptosis, and the suppression of PI3K/AKT/mTOR signaling in liver tumor (Carbajo-Pescador et al., 2014).

During WGCNA analysis, the blue module appeared to be the most relevant module involved in HB. The molecular function analysis revealed that genes in the blue module were enriched for the tubulin binding, microtubule binding, and microtubule motor activity pathways. Disrupted microtubule dynamics have previously been reported to modulate cell proliferation in several human tumors, including hepatocellular carcinoma (HCC) (Zhang et al., 2016; Aboubakr et al., 2017). The cellular component analysis revealed that genes in the blue module were predominantly associated with spindle, kinetochore, and mitotic spindle cellular components, which serve essential functions during mitosis (Sharp et al., 2000). Collectively, GO analysis showed that genes in the blue module were enriched for pathways such as organelle fission, cell cycle phase transition, chromosomal region, ATPase activity, catalytic activity, and acting on DNA. KEGG analysis indicated that genes in the blue module were enriched in the cell cycle, oocyte meiosis, and DNA replication pathways. Notably, the results from WGCNA GO/KEGG analysis were similar to the functional annotations of genes in the most significant module of PPI network.

The cell cycle is a set of organized and monitored stages through which a cell passes between cell divisions. Cells pass through the G0/G1, S, and G2 phases of interphase and subsequently directly enter the M phase, in which nuclear and cell division takes place (Norbury and Nurse, 1992). The progression from one stage of the cell cycle to another is controlled at checkpoints, which are regulated by interactions between cyclin-dependent kinases (CDKs) and their cyclin partners. Deregulation of the cell cycle may result in unscheduled proliferation, chromosome segregation defects, and ultimately the development of tumor (Bannon and Mc Gee, 2009). Indeed, cell cycle proteins are frequently overactive in tumor cells, and blocking cell cycle progression through inhibiting cell cycle proteins can lead to cell proliferation arrest in many tumor types. For instance, the retinoblastoma (Rb) gene encodes a tumor suppressor protein that is responsive to mitogenic signals to integrate the control of cell cycle (Hanahan and Weinberg, 2011). In tumor cells, defects in the Rb pathway give rise to the deregulation of the G1/S-phase cell cycle checkpoint, which in turn can lead to uncontrolled cell proliferation (Dyson, 1998). Using an approach combining bioinformatics analysis and subsequent experimental verification, we identified CDK1, CCNA2, and CDC20 as pivotal genes and potential biomarkers for future HB therapy. Interestingly, we found that all of these three key genes were involved in cell cycle (Figures 3E, 5D, 6F,G).

CCNA2 has previously been reported to be associated with chromosomal instability, epithelial–mesenchymal transition (EMT), and metastasis in tumors (Cheung et al., 2015). Specifically, CCNA2 binds to and activates CDK1 and CDK2, resulting in the formation of CDK/CCNA2 complex. It has been demonstrated that the CDK/CCNA2 complex drives S-phase progression (Girard et al., 1991; Yam et al., 2002), persists through the S and G2 phases, and is degraded upon entry into mitosis (den Elzen and Pines, 2001). Conversely, a decreased proliferative capacity of tumor cells has been observed after inhibition of the CDK/CCNA2 complex (Chen et al., 2004). Animal experiments indicated that a CCNA2 deficiency in hepatocytes may lead to the delayed formation of liver tumors (Gopinathan et al., 2014). At the cellular level, argininosuccinate lyase may promote HCC progression in association with CCNA2 (Hung et al., 2017). Our integrated microarray analysis revealed an upregulation of CCNA2 in HB tissues (Figure 8A), which is in line with results from a previous study (Shin et al., 2011). Moreover, *in vitro* experiments from our current study demonstrated, for the first time, that CCNA2 knockdown suppresses the proliferative, migrative, and invasive capacities of two HB cell lines.

In addition to regulation by CCNA2, the cell cycle is also modulated by CDKs via catalyzing phosphorylation of specific proteins (Ubersax et al., 2003). CDK1, one member of CDK family, is essential for mitosis, and inhibition of CDK1 has been shown to promote apoptosis in lymphomas and liver tumors in mice expressing MYC: in MYC-expressing HB transgenic mouse models, administration of a CDK1 inhibitor resulted in reduced tumor growth as well as extended survival (Goga et al., 2007). Taken together, these findings illustrate that CDK1 inhibition might specifically suppress the proliferative capacity of tumor cells. Similar to previous studies, in the present study, we demonstrate a significantly higher expression of CDK1 in HB tissue relative to normal liver tissue. Additionally, our functional assays indicated that CDK1 knockdown suppressed proliferative, migrative, and invasive properties of two HB cell lines.

In addition to CCNA2 and CDK1, our study also identified CDC20 as a key hub gene involved in HB growth, and subsequent experiments further demonstrated that aggressive biological behaviors of HB cell lines were inhibited after CDC20 knockdown. Previous studies have reported aberrantly high expression levels of CDC20 in oral squamous cell carcinoma (Mondal et al., 2007), gastric cancer (Kim et al., 2005), and lung adenocarcinoma (Liu et al., 2018). CDC20 knockdown has been shown to contribute to G2/M arrest, inhibiting tumor cell cycle progression (Kidokoro et al., 2008). Collectively, exploring therapeutic agents targeting the cell cycle via inhibition or modulation of CDK1, CCNA2, or CDC20 may be considered a promising therapeutic strategy for HB. In a recent study, Aghajanzadeh et al. (2020) identified 15 hub genes involved in HB based on bioinformatics analysis of GSE131329. CDK1 and CCNA2 were identified as hub genes in their study while CDC20 was not. This discrepancy could be due to the fact that different analytic methods were used and distinct datasets were assessed between their study and ours. Interestingly, using gene set enrichment and pathway analysis of the hub genes, the authors also identified cell cycle events as essential processes for HB development, which is in line with our findings.

The current study has some limitations. Experimental verification was only conducted *in vitro* at cellular level. In addition, the sample sizes for HB and normal liver tissue samples were asymmetrical, which may have potentially introduced bias in our analysis.

In conclusion, we conducted an integrative analysis of large-scale microarray gene expression profiling followed by experimental validation to investigate potential biomarkers and key genes involved in HB pathogenesis. By utilizing both PPI and WGCNA analyses, we identified CCNA2, CDK1, and CDC20 as hub genes in human HB. Subsequent *in vitro* experiments validated a potential oncogenic role for these three hub genes in two HB cell lines. Collectively, CCNA2, CDK1, and CDC20 may serve as promising biomarkers for HB and provide prospects for designing targeted therapies using synthetic inhibitors as anti-tumor agents.

## Data Availability Statement

The original contributions presented in this study are included in the article/Supplementary Material.

## Ethics Statement

The medical research ethics committee of Basic Medicine School, Guilin Medical University identified and approved this study design.

## Author Contributions

YZ and SD created the study design. LT, TC, YZ, and JL performed the experiments and data analyses. LT, TC, PQ, and JY wrote the manuscript. All authors contributed to the article and approved the submitted version.

## Conflict of Interest

The authors declare that the research was conducted in the absence of any commercial or financial relationships that could be construed as a potential conflict of interest.

## Footnotes

1 https://www.ncbi.nlm.nih.gov/geo/

## Supplementary Material

Supplementary Figure 1 Hierarchical clustering tree of samples and application of soft-threshold powers. **(A)** Hierarchical clustering of samples to detect outliers. **(B)** After network topology analysis for soft-threshold powers, the scale-free topology β = 10 was determined as soft threshold power. **(C)** Scale-free fitting exponential analysis of various soft threshold powers.
