# Supplementary material for: Integrated Protein–Protein Interaction and Weighted Gene Co-expression Network Analysis Uncover Three Key Genes in Hepatoblastoma
Source: Front Cell Dev Biol. 2021 Feb 26;9:631982. doi: 10.3389/fcell.2021.631982 (PMC7953069; doi:10.3389/fcell.2021.631982)
Supplement: Supplementary file 1 [file Table_1.DOCX]

**Table S1. Top 100 genes of the WGCNA blue module** **ranked by gene significance with cancer**

| Gene Symbol | Entrez ID | GS | *p*.GS | MM | *p*.MM |
| --- | --- | --- | --- | --- | --- |
| TYMS | 7298 | 0.760462333 | 4.11E-23 | 0.873173539 | 2.24E-37 |
| CCNB2 | 9133 | 0.700752464 | 2.01E-18 | 0.943300209 | 1.99E-56 |
| GSDME | 1687 | 0.686258042 | 1.87E-17 | 0.7773358 | 1.08E-24 |
| PTPN14 | 5784 | 0.676969692 | 7.29E-17 | 0.732974295 | 8.49E-21 |
| GINS1 | 9837 | 0.673581359 | 1.18E-16 | 0.877950242 | 2.89E-38 |
| PAPSS1 | 9061 | 0.669796875 | 2.02E-16 | 0.794009126 | 2.13E-26 |
| NAP1L1 | 4673 | 0.666620025 | 3.14E-16 | 0.78855674 | 7.98E-26 |
| MCM3 | 4172 | 0.663592451 | 4.75E-16 | 0.895541759 | 6.76E-42 |
| PFKM | 5213 | 0.660897974 | 6.86E-16 | 0.732027502 | 1.01E-20 |
| NCAPD2 | 9918 | 0.658326898 | 9.69E-16 | 0.819148397 | 2.74E-29 |
| SUPT3H | 8464 | 0.656899889 | 1.17E-15 | 0.768130723 | 8.16E-24 |
| MELK | 9833 | 0.654904437 | 1.53E-15 | 0.935574408 | 2.32E-53 |
| HMGA2 | 8091 | 0.647088179 | 4.23E-15 | 0.875061863 | 1.01E-37 |
| CDK1 | 983 | 0.644067743 | 6.22E-15 | 0.950176392 | 1.52E-59 |
| GSTA4 | 2941 | 0.642730369 | 7.37E-15 | 0.681228221 | 3.93E-17 |
| CDKN3 | 1033 | 0.642298915 | 7.78E-15 | 0.927722361 | 1.30E-50 |
| FOXM1 | 2305 | 0.641637979 | 8.46E-15 | 0.878927333 | 1.88E-38 |
| SLC26A2 | 1836 | 0.638088896 | 1.32E-14 | 0.796484344 | 1.15E-26 |
| TOP2A | 7153 | 0.634776141 | 1.98E-14 | 0.945131032 | 3.22E-57 |
| MCM7 | 4176 | 0.634249936 | 2.12E-14 | 0.81700568 | 5.02E-29 |
| RFC3 | 5983 | 0.623412281 | 7.78E-14 | 0.886412661 | 6.15E-40 |
| MCM2 | 4171 | 0.62067071 | 1.07E-13 | 0.835398273 | 2.08E-31 |
| AFP | 174 | 0.616470334 | 1.74E-13 | 0.754176777 | 1.48E-22 |
| MCM6 | 4175 | 0.613727941 | 2.39E-13 | 0.947833962 | 1.95E-58 |
| CCNA2 | 890 | 0.61246697 | 2.75E-13 | 0.93483276 | 4.36E-53 |
| H2AZ1 | 3015 | 0.610746942 | 3.34E-13 | 0.906416126 | 1.76E-44 |
| RPS7 | 6201 | 0.610717261 | 3.36E-13 | 0.809850978 | 3.60E-28 |
| MAP4K4 | 9448 | 0.605821915 | 5.80E-13 | 0.773063224 | 2.79E-24 |
| VPS72 | 6944 | 0.605541286 | 5.98E-13 | 0.582503217 | 6.92E-12 |
| OLR1 | 4973 | 0.603255849 | 7.69E-13 | 0.653657578 | 1.80E-15 |
| MSH2 | 4436 | 0.599878869 | 1.11E-12 | 0.90492749 | 4.14E-44 |
| PTTG1 | 9232 | 0.597677224 | 1.41E-12 | 0.907879909 | 7.47E-45 |
| KNTC1 | 9735 | 0.595388422 | 1.80E-12 | 0.844476774 | 1.08E-32 |
| ZWINT | 11130 | 0.595371656 | 1.81E-12 | 0.906960043 | 1.28E-44 |
| CCT3 | 7203 | 0.589723007 | 3.28E-12 | 0.765347329 | 1.48E-23 |
| ANLN | 54443 | 0.585587123 | 5.04E-12 | 0.909211569 | 3.38E-45 |
| STIL | 6491 | 0.585335795 | 5.17E-12 | 0.938391147 | 1.97E-54 |
| ILF2 | 3608 | 0.585263316 | 5.21E-12 | 0.841835457 | 2.60E-32 |
| MCM5 | 4174 | 0.581742644 | 7.48E-12 | 0.676360582 | 7.95E-17 |
| PCNA | 5111 | 0.581386223 | 7.75E-12 | 0.869564329 | 9.97E-37 |
| KIFC1 | 3833 | 0.581010661 | 8.06E-12 | 0.742842116 | 1.35E-21 |
| CKAP2 | 26586 | 0.578406032 | 1.05E-11 | 0.679929165 | 4.75E-17 |
| RRM2 | 6241 | 0.577919064 | 1.10E-11 | 0.901116018 | 3.49E-43 |
| DTYMK | 1841 | 0.57390199 | 1.64E-11 | 0.783006705 | 2.95E-25 |
| SUV39H2 | 79723 | 0.571363391 | 2.11E-11 | 0.890603562 | 8.15E-41 |
| ADAM9 | 8754 | 0.570466357 | 2.31E-11 | 0.696094226 | 4.18E-18 |
| MRPL9 | 65005 | 0.56759661 | 3.05E-11 | 0.744677545 | 9.54E-22 |
| KPNA2 | 3838 | 0.566751642 | 3.32E-11 | 0.886942475 | 4.79E-40 |
| RCN2 | 5955 | 0.560887225 | 5.82E-11 | 0.834929503 | 2.41E-31 |
| TMEM237 | 65062 | 0.560695215 | 5.93E-11 | 0.897905326 | 1.96E-42 |
| ZNF382 | 84911 | 0.55995957 | 6.35E-11 | 0.641089226 | 9.06E-15 |
| CDC20 | 991 | 0.559246399 | 6.80E-11 | 0.851202164 | 1.06E-33 |
| CETN3 | 1070 | 0.555403785 | 9.75E-11 | 0.702935319 | 1.42E-18 |
| SNRPE | 6635 | 0.553758242 | 1.14E-10 | 0.783769393 | 2.47E-25 |
| POLE2 | 5427 | 0.553171142 | 1.20E-10 | 0.88533466 | 1.02E-39 |
| CENPF | 1063 | 0.553022649 | 1.22E-10 | 0.928645364 | 6.43E-51 |
| IGF2BP3 | 10643 | 0.552741911 | 1.25E-10 | 0.83961748 | 5.37E-32 |
| CHEK1 | 1111 | 0.552321796 | 1.30E-10 | 0.929816668 | 2.59E-51 |
| PCLAF | 9768 | 0.551972989 | 1.34E-10 | 0.858458064 | 7.58E-35 |
| AURKA | 6790 | 0.549804033 | 1.64E-10 | 0.850457565 | 1.37E-33 |
| TMEM106C | 79022 | 0.547858311 | 1.95E-10 | 0.75488862 | 1.28E-22 |
| HUNK | 30811 | 0.54723539 | 2.07E-10 | 0.83423062 | 3.01E-31 |
| ATIC | 471 | 0.544862799 | 2.56E-10 | 0.813288844 | 1.41E-28 |
| LAPTM4B | 55353 | 0.543656161 | 2.86E-10 | 0.80343309 | 1.96E-27 |
| SMC3 | 9126 | 0.541536354 | 3.45E-10 | 0.663177201 | 5.03E-16 |
| TK1 | 7083 | 0.540509844 | 3.78E-10 | 0.665828409 | 3.50E-16 |
| RAD51 | 5888 | 0.538480368 | 4.53E-10 | 0.86272067 | 1.50E-35 |
| CDC6 | 990 | 0.538456762 | 4.54E-10 | 0.896677482 | 3.75E-42 |
| PRKDC | 5591 | 0.535765264 | 5.74E-10 | 0.85359089 | 4.51E-34 |
| FAM216A | 29902 | 0.528977354 | 1.03E-09 | 0.735458225 | 5.39E-21 |
| SPINK1 | 6690 | 0.528635608 | 1.06E-09 | 0.747809329 | 5.21E-22 |
| HDAC2 | 3066 | 0.527059182 | 1.22E-09 | 0.841597224 | 2.81E-32 |
| CDC45 | 8318 | 0.526658976 | 1.26E-09 | 0.846636383 | 5.17E-33 |
| CCNF | 899 | 0.525619546 | 1.37E-09 | 0.789939282 | 5.73E-26 |
| MAD2L1 | 4085 | 0.524906809 | 1.46E-09 | 0.949748003 | 2.45E-59 |
| EZH2 | 2146 | 0.522418886 | 1.80E-09 | 0.907704329 | 8.28E-45 |
| CDC25C | 995 | 0.522341902 | 1.81E-09 | 0.883612981 | 2.27E-39 |
| BARD1 | 580 | 0.522159164 | 1.84E-09 | 0.783966181 | 2.36E-25 |
| DLGAP5 | 9787 | 0.522094566 | 1.85E-09 | 0.942158738 | 5.99E-56 |
| PLK4 | 10733 | 0.520578186 | 2.10E-09 | 0.877451295 | 3.59E-38 |
| KIF11 | 3832 | 0.519509242 | 2.29E-09 | 0.942534505 | 4.18E-56 |
| TTK | 7272 | 0.51718456 | 2.78E-09 | 0.943412466 | 1.78E-56 |
| SPC25 | 57405 | 0.517135608 | 2.79E-09 | 0.889776706 | 1.22E-40 |
| C2orf15 | 150590 | 0.516457407 | 2.95E-09 | 0.667441086 | 2.80E-16 |
| RNASEH2A | 10535 | 0.51550389 | 3.19E-09 | 0.806581442 | 8.61E-28 |
| RAN | 5901 | 0.513215531 | 3.84E-09 | 0.87954002 | 1.43E-38 |
| RBL1 | 5933 | 0.513117192 | 3.88E-09 | 0.823602981 | 7.55E-30 |
| TRIP13 | 9319 | 0.512269105 | 4.15E-09 | 0.891867464 | 4.36E-41 |
| IRAK1BP1 | 134728 | 0.51131498 | 4.48E-09 | 0.753709293 | 1.62E-22 |
| EXO1 | 9156 | 0.509428081 | 5.22E-09 | 0.920854057 | 1.89E-48 |
| C17orf75 | 64149 | 0.508757688 | 5.51E-09 | 0.68836584 | 1.36E-17 |
| TPX2 | 22974 | 0.508325806 | 5.70E-09 | 0.899596295 | 7.96E-43 |
| ETV5 | 2119 | 0.507254982 | 6.21E-09 | 0.678576864 | 5.78E-17 |
| PRTG | 283659 | 0.507093461 | 6.29E-09 | 0.544875465 | 2.56E-10 |
| SGO1 | 151648 | 0.50311813 | 8.62E-09 | 0.617546633 | 1.54E-13 |
| CBX5 | 23468 | 0.499476563 | 1.15E-08 | 0.669033749 | 2.24E-16 |
| PCSK5 | 5125 | 0.499347247 | 1.16E-08 | 0.736523597 | 4.43E-21 |
| H2AX | 3014 | 0.498697683 | 1.22E-08 | 0.708298769 | 5.97E-19 |
| RAD51AP1 | 10635 | 0.4984516 | 1.24E-08 | 0.929997702 | 2.25E-51 |
| MTMR2 | 8898 | 0.497874555 | 1.30E-08 | 0.722698933 | 5.28E-20 |

Abbreviations: GS: gene significance with cancer; MM: module membership; *p*.GS: *p* value of gene significance with cancer; *p*.MM: *p* value of module membership; WGCNA: weighted gene co-expression network analysis.
