# Supplementary figures and images for: Integrated Protein–Protein Interaction and Weighted Gene Co-expression Network Analysis Uncover Three Key Genes in Hepatoblastoma
Source: Front Cell Dev Biol. 2021 Feb 26;9:631982. doi: 10.3389/fcell.2021.631982 (PMC7953069; doi:10.3389/fcell.2021.631982)

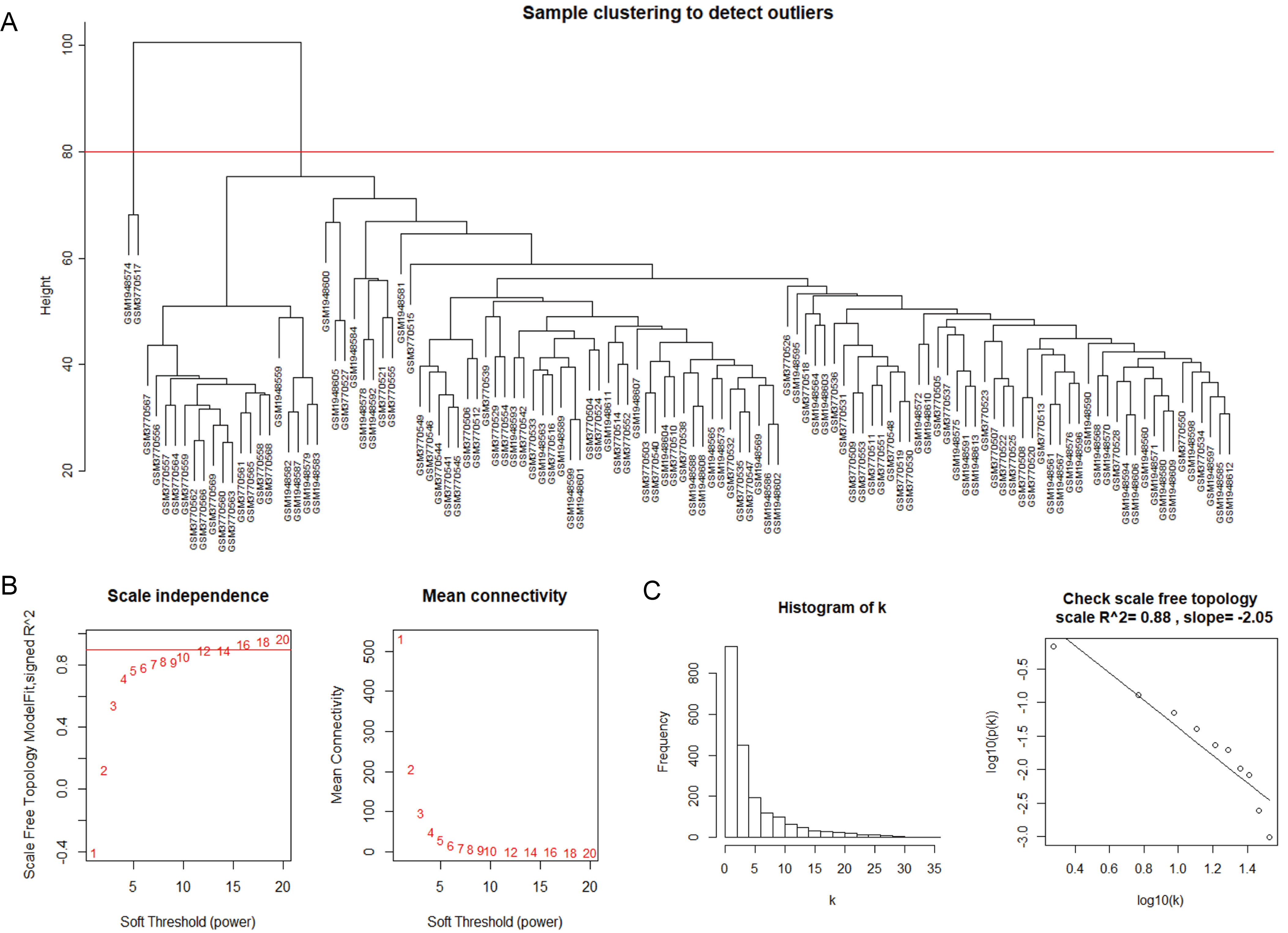

Supplement: Supplementary Figure 1 — Hierarchical clustering tree of samples and application of soft-threshold powers. (A) Hierarchical clustering of samples to detect outliers. (B) After network topology analysis for soft-threshold powers, the scale-free topology β = 10 was determined as soft threshold power. (C) Scale-free fitting exponential analysis of various soft threshold powers. [file Image_1.TIF]
